# Development of a Binderless Particleboard from Brown Seaweed *Sargassum* spp.

**DOI:** 10.3390/ma17030539

**Published:** 2024-01-23

**Authors:** Jérôme Bauta, Guadalupe Vaca-Medina, Christine Delgado Raynaud, Valérie Simon, Virginie Vandenbossche, Antoine Rouilly

**Affiliations:** 1 Laboratoire de Chimie Agro-Industrielle (LCA), Université de Toulouse, INRAE, Toulouse INP, 31030 Toulouse, France; 2 Centre d’Application et de Traitement des Agro-Ressources (CATAR), Toulouse-INP, 4 allée Emile Monso, 31030 Toulouse, France

**Keywords:** *Sargassum natans*, *Sargassum fluitans*, thermo-compression, binderless particleboard, alginates

## Abstract

Since 2010, huge quantities of *Sargassum* spp. algae have been proliferating in the Atlantic Ocean and stranding on Caribbean beaches, causing major economic, environmental, and health problems. In this study, an innovative high-density binderless particleboard was developed using uniaxial thermo-compression coupled with a cooling system. The raw material consisted of ground *Sargassum* seaweeds pre-treated by twin-screw extrusion with water to remove sea salt. The raw material and the particleboards were produced by using various analytical techniques such as Dynamic Vapor Sorption (DVS), Differential Scanning Calorimetry (DSC), Dynamic Mechanical Analysis (DMA), or Thermogravimetric Analysis (TGA). The experimental conditions for thermo-compression (temperature, pressure, time) were evaluated. The best thermo-compression conditions tested were 200 °C, 40 MPa pressure for 7.5 min. This resulted in a particleboard with high density (up to 1.63 ± 0.02 g/cm^3^) and high flexural strength/modulus (up to 32.3 ± 1.8 MPa/6.8 ± 0.2 GPa, respectively), but a low water contact angle of 38.9° ± 3.5°. Thermal analyses revealed the effect of alginates on the mechanical properties of particleboards. This work opens the door to a new way of adding value to *Sargassum* seaweed, using the whole algae with minimal pre-treatment.

## 1. Introduction

For the past decade, *Sargassum* spp. seaweeds have been a major problem for the Caribbean coastline. Originally restricted to the Sargasso Sea, since 2011, they have colonized a large part of the Atlantic Ocean, forming a belt between West Africa and South America, more than 8000 km long [1]. Every year, the Caribbean coasts experience massive strandings between January and September. In the record year of 2018, for example, more than 3.2 × 10^3^ m^3^/km·month washed up on the Mexican coast [2]. Similarly, the island of Guadeloupe has estimated that between 20,000 and 50,000 tons (dry matter basis) of seaweeds are deposited on its coasts every year [3].

These strandings cause health, economic, and environmental problems for local populations. Beach-cast seaweeds start to decompose, releasing sulphurous gases (H_2_S in particular) and ammonia [3]. Local residents are exposed both acutely and chronically, which can sometimes lead to serious symptoms such as abdominal pain, respiratory problems, eye and skin disorders, or headaches [4].

The decomposition of algae in the water releases particles of organic matter and leachates, leading to a series of harmful effects. These include reduced luminosity under *Sargassum* mats, increased turbidity, changes in water color, and hypoxia [5]. These effects lead to the death of fish, crustaceans, seagrasses, and corals, as well as to a reduction in their biodiversity [5,6,7]. The strandings themselves lead to accelerated beach erosion, accentuated by the mechanical gathering of beach-cast seaweed [8].

The impact of seaweed collection is significant in financial terms: some of the areas affected are heavily dependent on tourism. Mexico, for example, has invested 17 million dollars in the collection of seaweed [9]. Some hotels are also spending large sums to install barriers, purchase machinery and boats, and transport them by truck [2]. Studies have also shown the impact of the gases emitted by *Sargassum* algae on metal corrosion, a phenomenon which may affect household appliances of local residents [10].

A number of studies have been conducted to find ways of using this humongous mass of *Sargassum* seaweeds in the agricultural, construction, and energy sectors [11,12,13]. However, there are a number of problems associated with the seaweed composition, the most challenging being their heavy metal content [14,15]. In fact; alginates, one of the main structural polysaccharides of brown algae, are known to chelate heavy metals, particularly arsenic [16,17]. This property could be useful in the treatment of polluted water [18,19], but it also makes other valorization pathways, where heavy metals are proscribed, more complex.

Biopolymer-based materials constitute one possible way to recover the material. However, most studies into the conversion of *Sargassum* seaweeds into materials focus on using only certain components of the plant. Examples include the use of alginates for biodegradable films [20,21,22], which can be used in the packaging sector, cellulose and alginates for biocomposites [23], and the transformation of *Sargassum* into char for soil decontamination [24] or into ash for use as pozzolanic material [25]. In addition to collection, transport, and packaging, the extraction of compounds of interest may require numerous physical and chemical processing stages. Other studies or commercial products use whole algae as fillers in biocomposites to modulate their properties: in shoe soles [26], building blocks [27], paper [28], or asphalt [29], for example.

No work on the manufacture of rigid materials solely made from algae appears to have been published. In this study, the first example of rigid binderless particleboard composed exclusively of *Sargassum* was developed by uniaxial thermo-compression coupled with a cooling device that improves the overall properties of such materials. This method has already been used to produce films from minimally processed red algae [30] and has been used extensively to develop panels from a variety of lignocellulosic materials. However, it has yet to be used to produce dense thick materials from biomasses based on alginate and cellulose such as brown algae. The raw material was processed by twin-screw extrusion to remove most of its bound sea salt and heavy metals. The solid residue obtained was then physically processed by Dynamic Vapor Sorption (DVS) and Thermogravimetrical Analysis (TGA) in order to study its capacity to be transformed into a bio-based material. The operating conditions for thermo-compression were studied in terms of temperature, mold pressure, and compression time in order to maximize flexion modulus and flexural strength. The mechanical properties of the particleboards were measured by three-point flexural tests. In order to investigate the impact of alginates on the mechanical properties of particleboards, thermal analyses (DMA, DSC) were conducted. This study could represent a first step toward the usage of whole seaweed specimens to produce 100% *Sargassum* materials for a wide range of applications (construction, design, etc.).

## 2. Materials and Methods

### 2.1. Sample Preparation

*Sargassum* seaweeds were collected on a beach in Punta Cana, Dominican Republic, then roughly washed with fresh water and sundried before shipping. The raw seaweeds were then coarsely ground using an Electra VS 1 hammer mill (Electra, Poudenas, France), then sieved on a TN1800 screening machine (Ritec, Saint-Paul-Trois-Châteaux, France) with a 1 mm sieve size to remove the excess sand. Their dry matter, mineral matter, and alginate contents were determined in a previous work [31]. Commercial sodium alginates were purchased from Sigma-Aldrich (Saint-Louis, MO, USA) and used as received.

The raw material used for thermo-compression was obtained after the pre-treatment of raw seaweeds by twin-screw extrusion with an Evolum 25 Lab Extruder (Clextral, Firminy, France). This treatment was used to wash the seaweeds with water and take away as much sea salt and heavy metals as possible. The screw profile used in this study is presented in Figure S1 and is composed of three main zones: the feeding, mixing, and pressing zones. First, *Sargassum* seaweeds were added to the feeding zone at a rate of 1.40 ± 0.04 kg/h DW (Dry Weight), followed by the injection of water at a Liquid/Solid ratio of 2.0. The temperature was kept constant inside the extruder at 30 °C. In the mixing zone, the seaweeds and solvent were intimately mixed by two series of bilobed paddles. Finally, in the pressing zone, reverse pitched screws exerted a strong compression and shearing force, leading to the separation of a liquid phase by filtration. Both the bilobes and reverse pitched screws helped reduce and homogenize the size of the *Sargassum* particles.

This process also allowed to recover a solid residue referred to as the “extrudate”. The extrudate was then dried at 50 °C for 24 h and then equilibrated under controlled conditions (25 °C and a relative humidity (RH) of 50%) for at least 2 weeks until a constant weight was achieved.

### 2.2. Thermo-Compression

The materials specimens were obtained by uniaxial compression of the extrudate (or commercial alginate powder) using a MAPA50 PEI hydraulic press (Pinette Emideceau, Chalon sur Saône, France) equipped with induction-heating plates and a water-cooling system (Roctool, Le-Bourget-du-Lac, France). This system enabled the press to be heated and cooled quickly. A total of 20 g of extrudate were loaded without any additive into a room temperature, three-piece 7 cm × 7 cm square steel mold. The temperature, pressure applied to the mold, and compression time were evaluated as shown in Table 1, while a cooling time of 10 min was kept constant. The resulting binderless plate was then cut into 1 cm × 5 cm specimens which were equilibrated under controlled conditions as described above for at least 1 week for further analyses. Two plates were obtained for each experimental condition tested, making it possible to obtain at least 10 specimens for the mechanical tests.

### 2.3. Characterization of Extrudate and Materials

Physicochemical and thermal analyses were performed on both the *Sargassum* extrudate and the particleboards. The moisture and ash contents of the extrudate were determined following the French standards NF EN ISO 18134-2 and NF EN ISO 18122, respectively [32,33]. The particle size of the extrudate was determined with an AS 200 Basic vibratory sieve shaker (Retsch, Haan/Duesseldorf, Germany) equipped with different sieves (mesh sizes: 2000–1000–800–500–200–125 µm). Its apparent and tapped density were measured with a Densitap ETD-20 (Granuloshop, Chatou, France).

An elemental analysis was performed on the raw material and extrudate using the AETE-ISO platform (OSU OREME/Université de Montpellier, Montpellier, France) with an iCAP Q ICP-MS (Thermo Scientific, Waltham, MA, USA).

An adsorption isotherm for the extrudate was conducted with a DVS Advantage System (Surface Measurement System, Alperton, UK) using 15 different RH steps (from 0 to 95%), with a dm/dt of 2 × 10^−3^%/min.

A Thermogravimetric Analysis (TGA) of the extrudate was performed using a TGA 2 Star System (Mettler-Toledo, Columbus, OH, USA) under 20 mL/min nitrogen flow using the following cycle: from 25 °C to 500 °C at 5 °C/min, then from 500 °C to 900 °C at 20 °C/min. A Derivative Thermogravimetry Analysis (DTGA) was then conducted using the TGA curve. A Dynamic Mechanical Analysis (DMA) was performed on the extrudate, sodium alginate powder, and materials in single cantilever mode using a Tritec 2000 (Triton Technology, Nottinghamshire, UK) equipped with a liquid nitrogen cooling system. Measurements were performed from −50 °C to 150 °C at a heating rate of 2 °C/min, with a displacement of 50 μm and at oscillation frequencies of 1 Hz and 10 Hz. For powder samples, steel pockets were used to perform the DMA analysis. A Differential Scanning Calorimetry (DSC) analysis was performed on the extrudate, sodium alginate, and particleboards using a DSC 1 (Mettler-Toledo, Columbus, OH, USA) and 40 µL aluminum capsules. The temperature gradients were as follows: from 25 °C to 80 °C, from 80 °C to 25 °C, and from 25 °C to 150 °C at 20 °C/min under a 50 mL/min nitrogen flow.

Optical images of the extrudate and materials were obtained with a SMZ1500 binocular loupe (Nikon, Tokyo, Japan), a Hirox HRX01 digital optical microscope (Hirox, Tokyo, Japan), and a Redmi Note 9 pro phone (Xiaomi, Beijing, China). Scanning Electron Microscopy (SEM) images of particleboards were obtained with a Quanta 450 (FEI, Hillsboro, OR, USA), with a 130 Pa water vapor partial pressure in the chamber and a high voltage of 12.50 kV. The SE microscope was equipped with a 30 mm^2^ silicone drift detector for elemental analysis.

A three-point flexion test was performed on the specimens using a H5KT Benchtop tester (Tinius Olsen, Horsham, PA, USA). A one-way Analysis of Variance (ANOVA) was performed on the results using the XLSTAT 2014.5.03 software, associated with a Shapiro–Wilk test (α = 0.05) to check the normality of residual data, a Levene’s test on the median (α = 0.05) to assess homoscedasticity, and a Tukey’s HSD (Honestly Significant Difference) test for multiple comparisons. The density of the material was obtained by buoyancy in cyclohexane. A specimen was weighted in air and in cyclohexane and its density d_specimen_ was calculated using Formula (1):

(1) $$d_{specimen}=\frac{W_{a}\times \left(d_{c}-d_{a}\right)}{\left(W_{a}-W_{c}\right)\times corr}+\rho_{a}$$

where d_a_ and d_c_ are the densities of air and cyclohexane, respectively, W_a_ and W_c_ are the weights of the specimen in air and immerged in cyclohexane, respectively, corr is a corrective coefficient for hydrostatic thrust due to the device being equal to 0.99983, and ρ_a_ is the density of air. Five specimens were weighed for each experimental condition.

Finally, the wettability of particleboards was estimated by measuring the water contact angles with a DGD-MCAT-V8 (GBX Scientific LTD., Tallaght, Ireland) by averaging 10 measurements.

## 3. Results and Discussion

### 3.1. Characterization of the Raw Seaweeds and Extrudate

**Figure 1:**
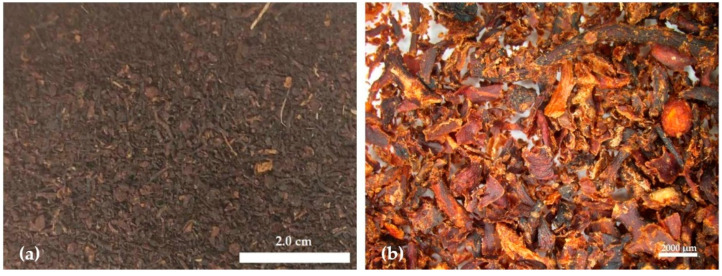
Picture (**a**) and binocular lens view (**b**) of the *Sargassum* extrudate.

The extrudate obtained by twin-screw extrusion was a solid fraction made up of inhomogeneous particles, as seen on Figure 1. Macroscopically, it showed no traces of sand or salt stuck to the particles. Its mineral content decreased from 36.5 ± 0.1 %DW to 26.5 ± 0.2 %DW. The ICP-MS analysis showed that the twin-screw extrusion extracted between 12.0% (Pb) and 51.1% (Na) of the elements analyzed, and, in particular, 25.0% of the cobalt, 25.9% of the cadmium, and 37.5% of the arsenic initially present in the algae (Figure S2). The apparent density of the extrudate was 0.39 ± 0.03 g/cm^3^, while its tapped density was 0.48 ± 0.03 g/cm^3^.

Extrusion has allowed a reduction in the granulometry of the seaweed: most of the raw seaweed was a few millimeters to a few centimeters in size, while the particles of extrudate were almost all less than 2 mm long. (Figure S3). Almost half of the particles were larger than a millimeter, while the other half were evenly distributed between 200 µm and 1000 µm. Visually, the largest particles appeared to be composed exclusively of blade and thallus fragments, while the finest fractions also included floats. For this study, the extrudate was not ground after extrusion in order to eliminate as many steps as possible in the development of the material. However, a review from Pintiaux et al. in 2015 showed that a fine and homogeneous particle size, generally, has a positive effect on mechanical properties of binderless particleboards [34].

**Figure 2:**
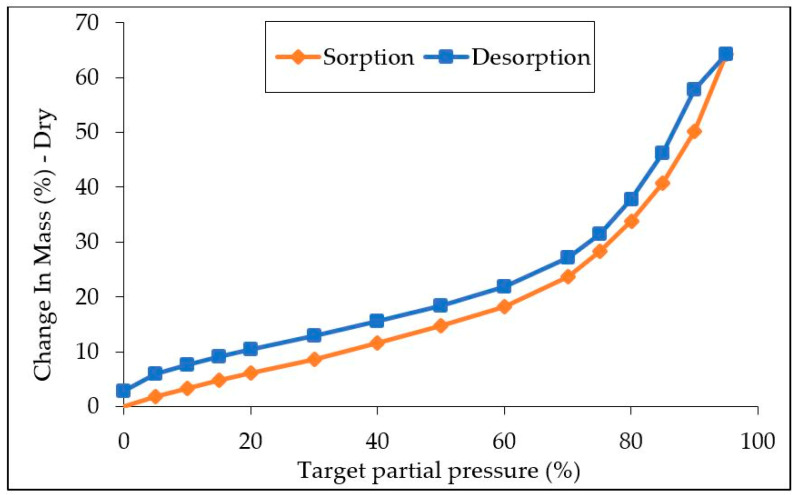
Water sorption and desorption isotherm of the *Sargassum* extrudate.

The sorption and desorption isotherms of water vapor for the extrudate are shown in Figure 2. The high hydrophilicity of the extrudate is immediately apparent. At 90% relative humidity, a mass gain of 64.2% is observed. By way of comparison, Simo-Tagne and coworkers showed that, for four types of exotic wood, the mass gain was only 16% to 18% at a relative humidity of 90% [35]. This tendency to uptake water may be linked to its alginate content: it has been shown that calcium alginates, for example, can uptake up to 200% of their initial weight in water at 90% RH [36]. Under the conditioning conditions applied to the *Sargassum* extrudate in this study prior to the thermo-compression (50% RH), a moisture content of 14.8% was reached.

**Figure 3:**
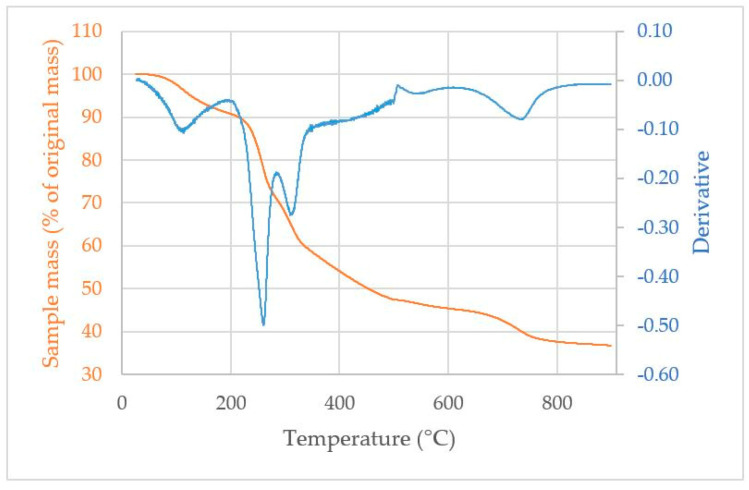
TGA (orange) and DTGA (blue) of the *Sargassum* extrudate.

Figure 3 represents the thermogravimetric analysis conducted on the extrudate. The result obtained is in agreement with the thermograms obtained by López-Aguilar et al. in 2020 on *Sargassum* spp. [37]. There are four peaks at 112 °C, 260 °C, 312 °C, and 735 °C for DTAG. The first peak corresponds to the volatilization of the water present in the extrudate. The second peak can be attributed to the decomposition of alginates, confirmed by numerous TGA and DSC studies of purified alginates [38] (Table 3), [39] (Tables 2 and 3). In particular, Soares et al. (2004) [40] noted an endothermic peak around 248 °C in a DSC analysis of sodium alginates. The third peak may be linked to the major devolatilization step in fucoidans described by Matusiak et al. [41]. Kristanto et al. also showed, using DSC and TGA, that cellulose exhibited a degradation peak at around 330 °C [42]. The loss of mass at higher temperatures can be attributed to the decarbonation of alginates forming different species (Na_2_CO_3_, CaCO_3_…) and, then, to their own decarbonation, leaving ashes rich in Na_2_O, CaO, and metals [37,39].

### 3.2. Study of Thermo-Compression Experimental Parameters of Sargassum Extrudate

**Figure 4:**
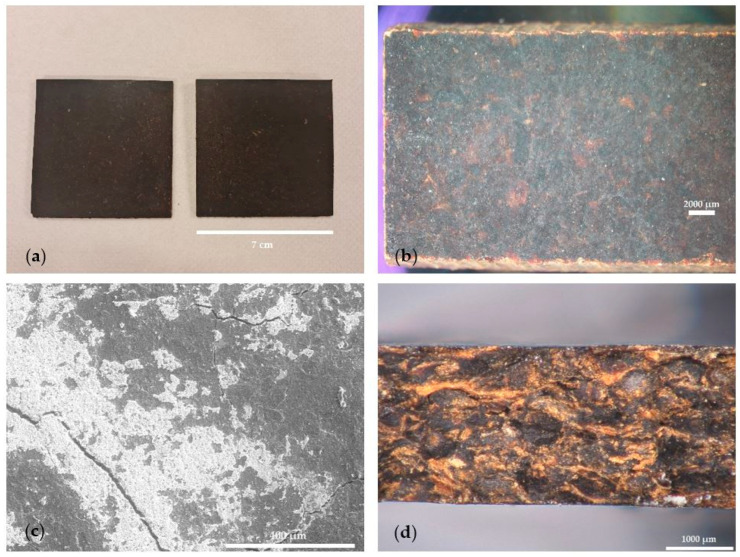
*Sargassum* particleboards images: photograph of plates (**a**), binocular lens view (**b**), SEM image of the surface of a test specimen (**c**), and digital optical view of its fracture point (**d**).

For the thermo-compression stage, the compression temperature and duration ranges were chosen to ensure the homogeneity of the particleboard obtained. With temperatures and times outside these ranges, the boards were visually undercooked or overcooked, and their structural integrity was not maintained during demolding or handling. The materials obtained by thermo-compression were, for the most part, smooth and homogeneous, as shown in the photographs in Figure 4. No particles were discernible either on the surface or on the edge of the materials, except for under the mildest applied conditions. The plates were between 2.5 mm and 3.0 mm thick in all conditions. The particles observed on the surface by MEB were grains of potassium chloride KCl, as shown in Figure S4. High levels of KCl have already been reported by Milledge et al. in the ashes of *Sargassum* samples [43]. However, no explanation was given in this study for these high levels. The presence of KCl crystals on the surface of the sample could itself be explained by the evaporation of the water contained in the extrudates on the surface of the material during compression [34].

**Figure 5:**
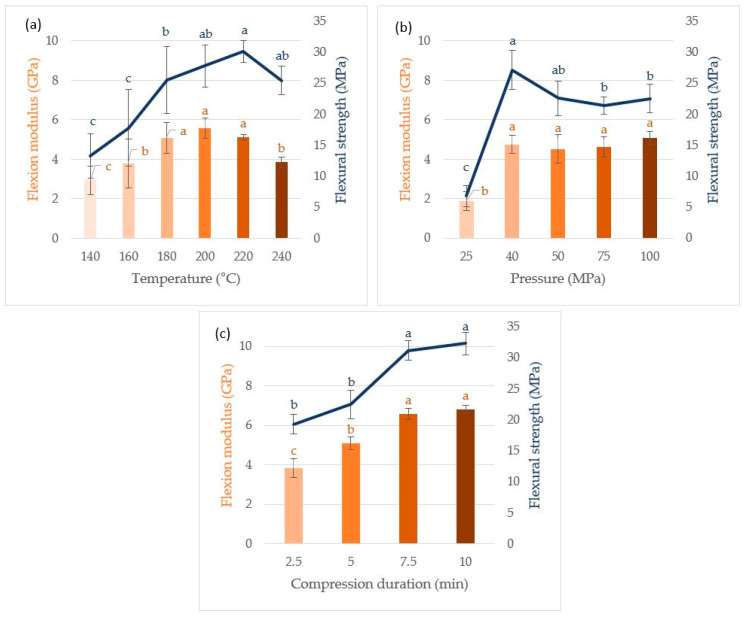
Influence of temperature (**a**), pressure (**b**), and compression time (**c**) on the flexion modulus and flexural strength of *Sargassum* particleboards. The other parameters used in this study are as follows: 100 MPa for 5 min (**a**), 180 °C for 5 min (**b**), 100 MPa at 180 °C (**c**). a–c letters on the graphs letters refer to Tukey’s HSD test.

Figure 5 represents the flexion modulus and flexural strength of the specimens obtained while varying the temperature (a), applied pressure (b), and compression time (c). The results obtained ranged from 1.9 ± 0.5 GPa/6.8 ± 1.7 MPa for flexion modulus/flexural strength (conditions: 180 °C, 25 MPa of pressure, 5 min) to a maximum of 6.8 ± 0.2 GPa/32.3 ± 1.8 MPa (conditions: 180 °C, 100 MPa of pressure, 10 min).

With regard to temperature, there was an increase in flexural modulus up to 200 °C and an increase in flexural strength up to 220 °C, reaching 5.6 ± 0.5 GPa and 30.1 ± 1.7 MPa, respectively. The results obtained are consistent with the thermogravimetric analyses conducted on the extrudate. Indeed, alginates, one of the main structural polymers present in algae, begin to degrade at around 200 °C, with a peak at 260 °C. An excessive degradation of the alginate matrix above 220 °C could explain the reduction in the mechanical properties of particleboard above this temperature. However, the partial degradation of alginates and other seaweed polysaccharides can lead to the formation of simple sugars and short-chain carbohydrates [44,45]. These sugars and carboxylic acids could both recombine under high temperatures and pressures and act as intrinsic binders between biopolymers, in particular by hydrogen bonding. This phenomenon has already been described as “self-bonding” by others [44,46].

Regarding the applied pressure, a large difference was visible between the low values at 25 MPa and the plateau reached from 40 MPa, resulting in a flexural modulus between 4.5 ± 0.7 GPa and 5.1 ± 0.3 GPa and a flexural strength between 21.4 ± 1.4 MPa and 27.1 ± 3.1 MPa. The mechanical properties also increased with the compression time, reaching 6.8 ± 0.2 GPa and 32.3 ± 1.8 MPa of flexural modulus/flexural strength for 10 min of compression. However, there was no clear difference between 7.5 min and 10 min of compression. Thus, a sufficiently high compression temperature seems necessary in order to activate the “self-bonding” mechanism and to allow the mobility of the carbohydrate chains, although an excessively elevated temperature would be detrimental on account of the degradation of the carbohydrate matrix of the Sargasso algae.

The materials obtained in this study generally exhibited flexural moduli and flexural strengths in the upper range of those obtained with other biomasses using the same molding process, although they have a different type of polysaccharide matrix than the common lignocellulosic system. It is worth mentioning the work of Uitterhaegen et al. on coriander cake resulting in a panel with a flexural modulus and flexural strength of 4.5 ± 0.4 GPa and 27.6 ± 1.3 MPa, respectively (experimental conditions: 205 °C, 21.6 MPa, 5 min) or that of Theng et al. resulting in a panel with higher mechanical characteristics (flexural modulus/flexural strength 8.6 ± 1.5 GPa/50.3 ± 9.0 MPa, experimental conditions: 200 °C, 22.3 MPa, 5 min) [47,48]. In comparison, a commercial pure sodium alginate material obtained in this study following the same process (experimental conditions: 180 °C, 100 MPa, 5 min) showed a higher flexural strength of 52.5 ± 4.2 MPa but a lower flexural modulus of 6.1 ± 0.1 GPa. These slightly weaker mechanical properties of *Sargassum*-based particleboards compared to boards made with pure alginate may, however, be balanced by the fewer extraction and processing steps required for their manufacture.

The wettability measurement conducted on the particleboard presenting the best mechanical properties in this study (experimental conditions: 200 °C, 100 MPa, 5 min) resulted in a water contact angle of 38.9° ± 3.5° just after the drop had been deposited (t = 0) and in the total disappearance of the drop after 60 s. This value is lower than that recorded for materials obtained from other biomasses. For example, Evon et al. found a water contact angle of between 70° and 90° at t = 0 and an angle of 35° after 160 s for a sunflower cake panel under the best conditions tested [49]. Danish et al. found angles between 90° and 110° at t = 0 and between 60° and 100° at t = 60 s for panels based on oil palm trunk and *Acacia mangium* wood trunks [50]. However, the low value in this study is consistent with the high water adsorption capacity of *Sargassum* extrudate reported by the DVS analysis.

The best conditions for thermo-compression of *Sargassum* extrudates in this study were found: a temperature of 200 °C, a pressure on the mold of 40 MPa, and a compression time of 7.5 min. This takes into account not only the results from the mechanical tests, but also the minimization of energy costs associated with the thermo-compression technique by the selection of milder experimental conditions (40 MPa of pressure instead of 100 MPa and 7.5 min of compression instead of 10 min). These conditions result in a particleboard with a flexion modulus that is equivalent to and a flexural strength that is only 38% inferior to the plate obtained with sodium alginate, even though the algal matrix is far more complex than pure alginates. This may show that it is possible to obtain particleboards based on *Sargassum* with high mechanical properties without going through a cumbersome, multi-stage alginate extraction process [51].

### 3.3. Impact of Experimental Conditions on Density

For the three experimental parameters studied, density seems to follow the same trend as that observed for the flexural modulus and maximum stress (Figure S5): with respect to temperature, an increase in density was observed up to 200 °C (reaching a maximum value of 1.63 ± 0.02 g/cm^3^) before a decrease; with respect to applied pressure, a plateau was reached after 40 MPa, leading to a density between 1.60 ± 0.02 g/cm^3^ and 1.62 ± 0.01 g/cm^3^. Finally, with respect to compression time, a plateau was also observed between 7.5 min and 10 min for a maximum density of 1.63 ± 0.01 g/cm^3^.

The improvement in mechanical properties could be directly related to the increase in material density. Indeed, Pintiaux et al. determined that a higher density within the material led to a higher contact surface between the particles [52]. As a result, more hydrogen bonds and Van der Waals forces can bind the particles together. Similarly, Okuda et al. found that, for lignocellulosic panels, the density influenced the chemical changes within the material (notably, the condensation of lignin in Okuda’s study) and, therefore, the mechanical strength of the binderless particleboard [53]. This could be mitigated by the thickness of the samples: samples obtained from a similar process with cellulose have shown orthotropic internal structures with sides and faces denser than the core of the specimens [54].

### 3.4. Role of Alginates during Thermo-Compression Process of Sargassum Particleboard

In order to try to explain the transformations within the material leading to the high mechanical properties of *Sargassum* particleboards, DMA and DSC analyses were conducted. Pure commercial sodium alginate, taken as the main component of the seaweed structural matrix [55], was also analyzed for comparison with *Sargassum* extrudate and its particleboard. The specimen used in these analyses was the one with the best mechanical properties (thermo-compression conditions: 180 °C, 100 MPa, 10 min).

**Figure 6:**
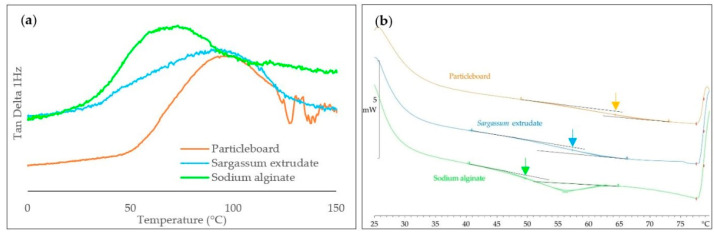
Evolution of Tan(δ) with temperature at 2 Hz (**a**) and DSC thermogram (first heating) (**b**) for sodium alginate powder (green), *Sargassum* extrudate (blue), and a *Sargassum* particleboard (orange).

The results for thermal analyses are shown in Figure 6. In the DMA study (Figure 6a), the evolution in modulus and loss factor Tan(δ) of the particleboard with temperature are also presented in Figure S6. The α-relaxation temperatures (T_α_) obtained, indicated by arrows on Figure 6b, were 73.0 °C, 92.2 °C, and 98.1 °C for sodium alginate, *Sargassum* extrudate, and particleboard, respectively. DMA analyses can provide valuable information to compare α-relaxation temperatures of powders and compressed materials. Powder analysis is specific, as it does not allow to measure modulus, but it is interesting for biopolymer thermal analysis, as it is very sensitive and show clearly the effect of water evaporation in this temperature range through the broadening of the peak in Tan(δ).

For the DSC analysis, only the first heating is presented (25 °C to 80 °C), as the other gradients (80 °C to 25 °C and 25 °C to 120 °C) showed no apparent transition, which means that this transition is non-reversible. The T_g_ observed was lower than the T_α_ presented previously, with 49.4 °C, 57.1 °C, and 64.2 °C for sodium alginate, *Sargassum* extrudate, and particle board, respectively. For sodium alginate, some physical aging is observed on the DSC trace, as it happens when purified biopolymers are stored in the glassy state. α-relaxation temperatures at 1 Hz correspond to the mechanical manifestations of the glass transition and are often observed at higher temperatures relative to T_g_ measurements by DSC. Also, a slight endothermic peak was observed for sodium alginate, probably corresponding to a relaxation of the polymer chains.

These analyses could demonstrate the impact of alginates as a thermoplastic matrix in the *Sargassum* seaweed complex structure during the production of binderless particleboards. First, a higher T_g_ was obtained for the *Sargassum* extrudate than for sodium alginate. This could indicate a matrix effect on chain mobility. In fact, as alginates interact with other polymer chains in the extrudate, they have less mobility and, therefore, need more energy to rearrange themselves. The T_g_ of the particleboard was also higher than those of the extrudate. This may be due to compression forcing new inter- and intra-biopolymer interactions that increase density, leading to a decrease in mobility between the chains and, once again, greater energy required to rearrange the polymer chains.

## 4. Conclusions

This study allowed to obtain the first binder-free particleboard based on whole *Sargassum* seaweeds by thermo-compression using twin-screw extrusion as a pre-treatment step. Various analytical methods were performed to examine the characteristics of the *Sargassum* extrudate. The best experimental parameters for thermo-compression in this study were as follows: a temperature of 200 °C, a mold pressure of 40 MPa, and a compression time of 7.5 min. The materials obtained through this process presented a high density and a relatively high flexural strength/modulus, but a low water contact angle explained by its high hydrophilic character due to alginate presence. The different transition temperatures obtained for pure sodium alginate, *Sargassum* extrudate and the particleboard showed the important role played by the thermoplastic behavior of alginates in the mechanical properties of the materials developed. Seaweed-based materials also showed a lower T_g_ and T_α_ than the extrudate and alginate powder because of their complex matrix and the interactions that take place between the different polysaccharides.

For future work, it may be possible to optimize the pre-treatment of the algae in order to influence the chemical form of the polysaccharides (alginate/alginic acid, for example) present in *Sargassum*, which could improve the mechanical properties of the resulting materials. In addition, these materials were determined to be very hydrophilic; this property may be of interest in applications where rapid degradation of materials is required. Otherwise, work could be conducted to improve the water resistance tests of *Sargassum* materials by developing treatment before or after thermo-compression, which could widen the possible range of applications. The preparation of materials by thermo-compression could nevertheless constitute an interesting way of using *Sargassum* seaweed for various industries (construction, design, etc.). As the processing of seaweed into materials presented in this study is fairly straightforward, companies in the areas most affected by strandings could easily develop new products based on this technology, for instance as biodegradable packaging.

## Figures and Tables

**Table 1:** Conditions studied for uniaxial thermo-compression of *Sargassum* extrudate.

	Temperature (°C)	Applied Pressure (MPa)	Compression Time (min)
Temperature	140–160–180–200–220–240	100	5
Pressure	180	25–40–50–75–100	5
Duration	180	100	2.5–5–7.5–10

## Data Availability

Data are contained within the article and supplementary materials.

## Supplementary Materials

The following supporting information can be downloaded at: https://www.mdpi.com/article/10.3390/ma17030539/s1, Figure S1: Screw profile used for *Sargassum* pre-treatment by twin screw extrusion; Figure S2: Elements extracted from raw seaweeds by twin-screw extrusion with water; Figure S3: Particle size distribution in the extrudate; Figure S4: Elemental analysis of the particles observed at the surface of a particleboard by SEM; Figure S5: Influence of Temperature, pressure on the mold and compression duration on the density of *Sargassum*-based material; Figure S6: DMA analysis of a particleboard obtained with the best experimental conditions.
