# Supplementary material for: Development of a Binderless Particleboard from Brown Seaweed Sargassum spp
Source: Materials (Basel). 2024 Jan 23;17(3):539. doi: 10.3390/ma17030539 (PMC10856414; doi:10.3390/ma17030539)
Supplement: Supplementary file 1 [file materials-17-00539-s001.zip › materials-2814169-supplementary.pdf]

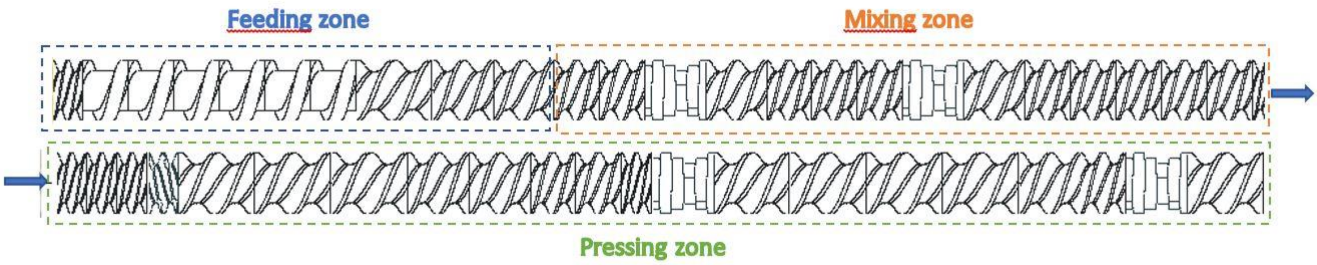

Figure S1: Screw profile used for *Sargassum* pre-treatment by twin screw extrusion

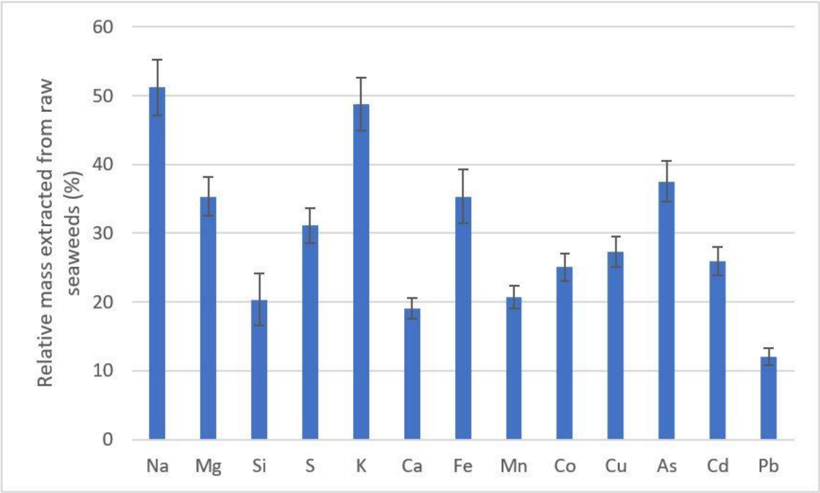

Figure S2: Elements extracted from raw seaweeds by twin-screw extrusion with water

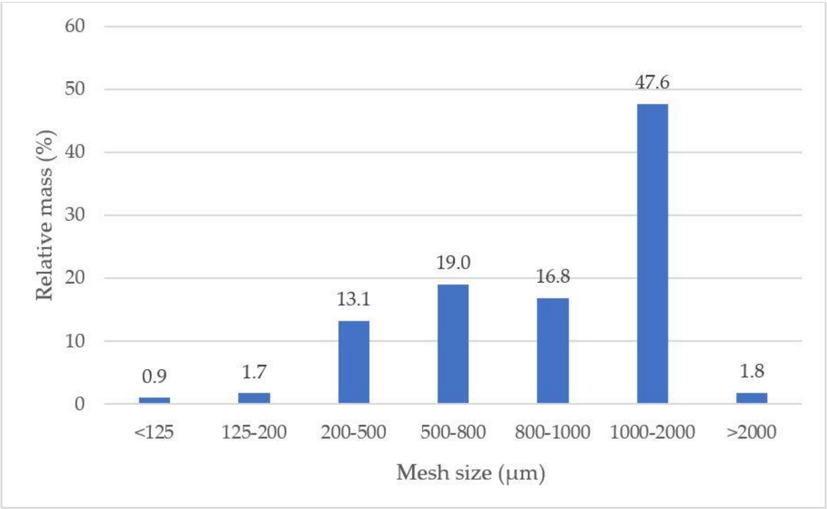

Figure S3: Particle size distribution in the extrudate

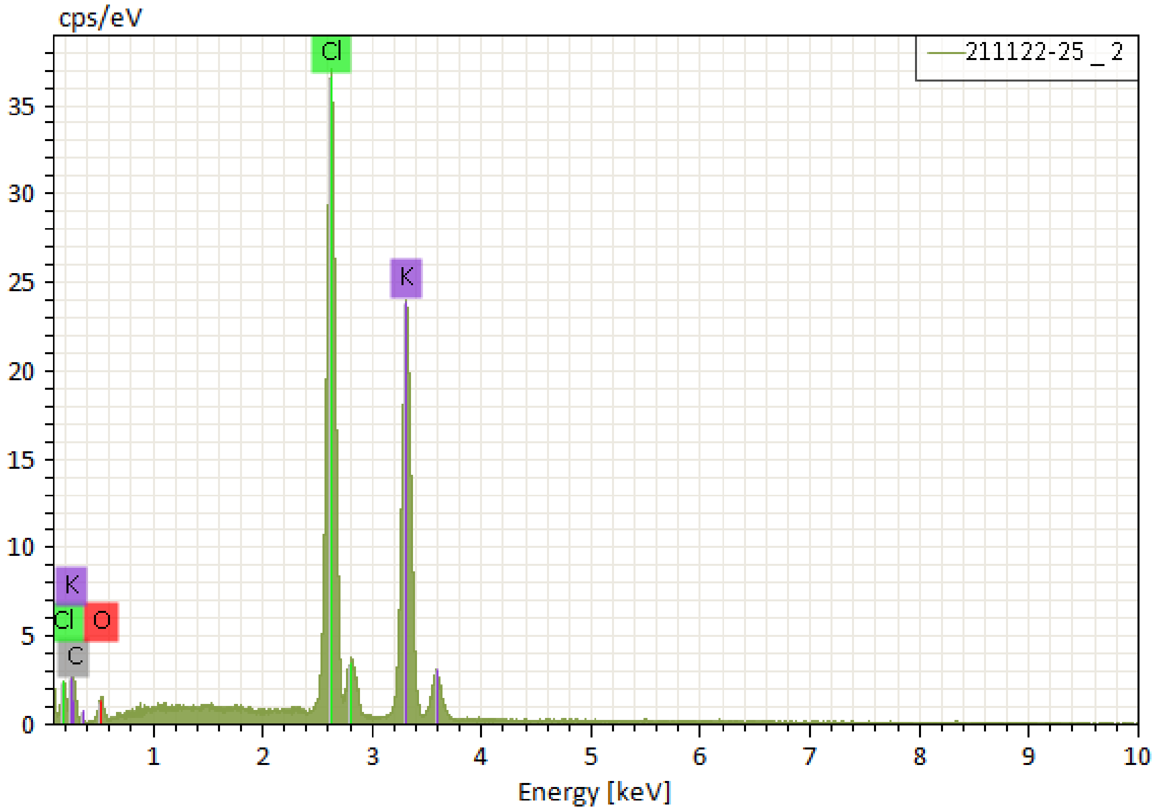

Figure S4: Elemental analysis of the particles observed at the surface of a particleboard by SEM

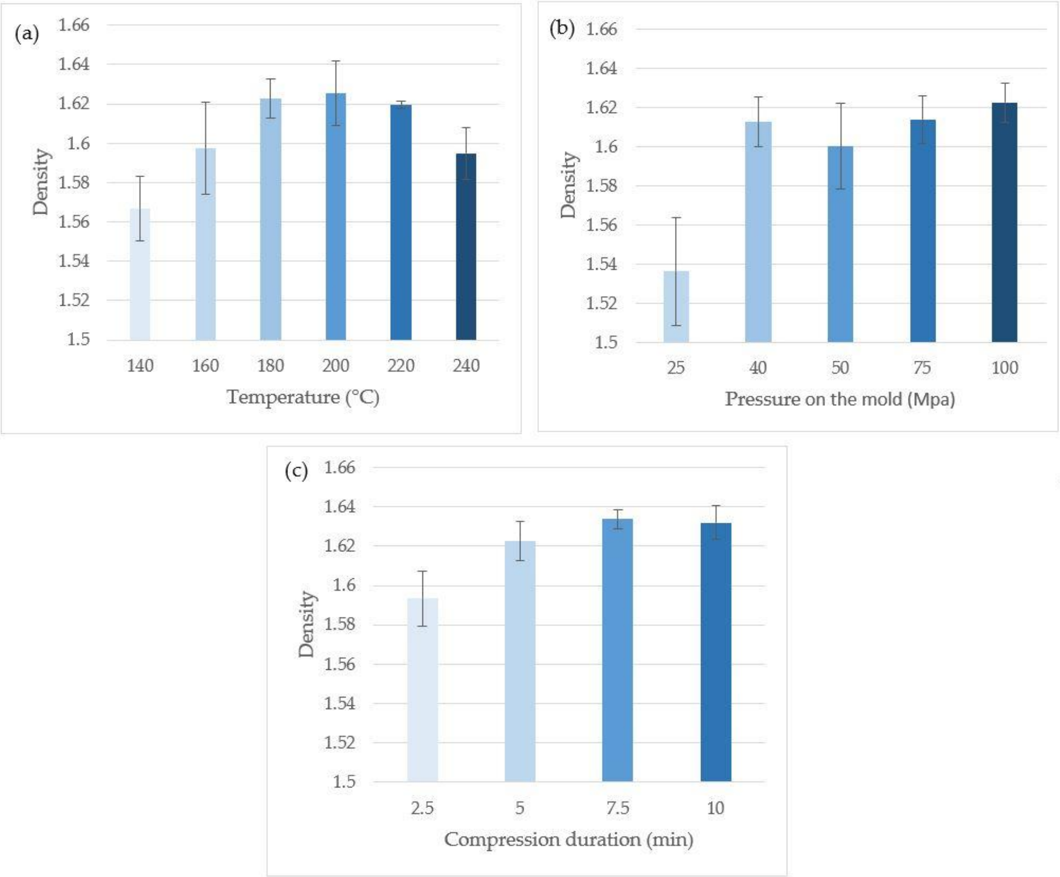

Figure S5: Influence of Temperature (a), pressure on the mold (b) and compression duration (c) on the density of *Sargassum*-based materials

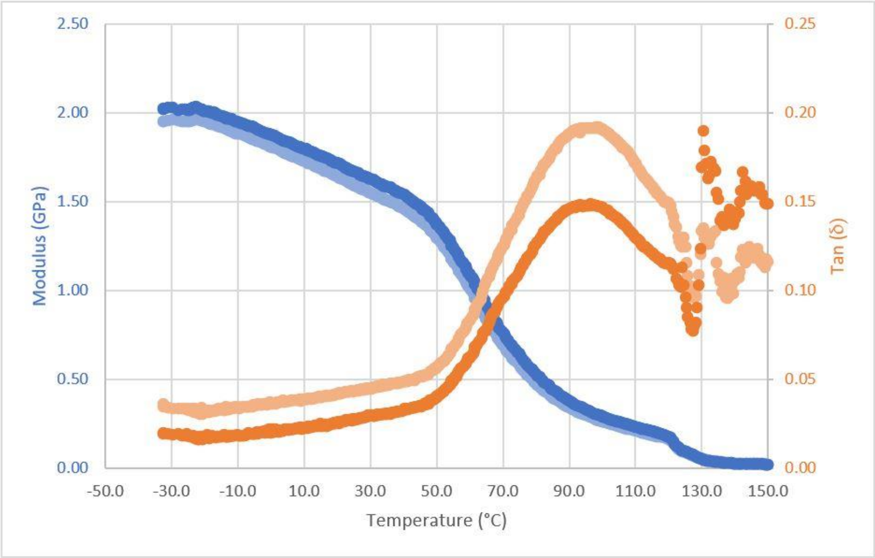

Figure S6: DMA analysis of a particleboard obtained with the best experimental conditions (200 °C, 100 Mpa, 7.5 min) (Lighter shade: 1 Hz oscillation, darker shade: 10Hz oscillation; blue: modulus, orange: loss modulus tan(δ))
